# COVID-19 Convalescent Plasma Is More than Neutralizing Antibodies: A Narrative Review of Potential Beneficial and Detrimental Co-Factors

**DOI:** 10.3390/v13081594

**Published:** 2021-08-11

**Authors:** Daniele Focosi, Massimo Franchini, Liise-anne Pirofski, Thierry Burnouf, DeLisa Fairweather, Michael J. Joyner, Arturo Casadevall

**Affiliations:** 1 North-Western Tuscany Blood Bank, Pisa University Hospital, 56124 Pisa, Italy; 2 Division of Transfusion Medicine, Carlo Poma Hospital, 46100 Mantua, Italy; massimo.franchini@asst-mantova.it; 3 Division of Infectious Diseases, Montefiore Medical Center, Albert Einstein College of Medicine, New York, NY 10467, USA; l.pirofski@einsteinmed.org; 4 Graduate Institute of Biomedical Materials and Tissue Engineering & International PhD Program in Biomedical Engineering, College of Biomedical Engineering, Taipei Medical University, Taipei 110, Taiwan; thburnouf@gmail.com; 5 Department of Cardiovascular Medicine, Mayo Clinic, Jacksonville, FL 32224, USA; fairweather.delisa@mayo.edu; 6 Department of Anesthesiology and Perioperative Medicine, Mayo Clinic, Rochester, MN 55905, USA; joyner.michael@mayo.edu; 7 Department of Medicine, Johns Hopkins School of Medicine, Johns Hopkins University, Baltimore, MD 21205, USA; acasade1@jhu.edu; 8 Department of Medicine, Johns Hopkins Bloomberg School of Public Health, Johns Hopkins University, Baltimore, MD 21205, USA

**Keywords:** convalescent plasma, antithrombin III, extracellular vesicles, ADAMTS13, MDA5, interferons, autoantibodies, controlled trials, decoy receptors, thrombosis, heterologous immunity

## Abstract

COVID-19 convalescent plasma (CCP) is currently under investigation for both treatment and post-exposure prophylaxis. The active component of CCP mediating improved outcome is commonly reported as specific antibodies, particularly neutralizing antibodies, with clinical efficacy characterized according to the level or antibody affinity. In this review, we highlight the potential role of additional factors in CCP that can be either beneficial (e.g., AT-III, alpha-1 AT, ACE2+ extracellular vesicles) or detrimental (e.g., anti-ADAMTS13, anti-MDA5 or anti-interferon autoantibodies, pro-coagulant extracellular vesicles). Variations in these factors in CCP may contribute to varied outcomes in patients with COVID-19 and undergoing CCP therapy. We advise careful, retrospective investigation of such co-factors in randomized clinical trials that use fresh frozen plasma in control arms. Nevertheless, it might be difficult to establish a causal link between these components and outcome, given that CCP is generally safe and neutralizing antibody effects may predominate.

## 1. Introduction

At the end of 2019, a novel flu-like coronavirus (CoV), named severe acute respiratory syndrome (SARS)-CoV-2 causing Coronavirus Disease 2019 (COVID-19), was associated with an epidemic initially focused on Wuhan, China. As a consequence of worldwide spread, COVID-19 was declared a pandemic by the World Health Organization (WHO, Geneva, Switzerland) on 11 March 2020 [1].

This new virus represented a major challenge for clinicians because it had no specific pre-existing therapy. Consequently, therapeutic efforts were initially focused on optimizing respiratory care, managing thrombotic and inflammatory complications using anticoagulation and corticosteroids, and repurposing existing antiviral therapies [2]. Unfortunately, nearly all these initially promising agents (i.e., hydroxychloroquine and lopinavir/ritonavir) showed limited clinical benefit [3]. Considering the lack of effective anti-SARS-CoV-2 drugs and the initial positive experience from China [4], convalescent plasma, old passive immunotherapy used with apparent success in many prior epidemics and outbreaks since the 1918 Spanish flu epidemic, was proposed for COVID-19. Several randomized and nonrandomized controlled trials were published last year, overall documenting that COVID-19 convalescent plasma (CCP), if administered at high-titer (>1:160 anti-SARS-CoV-2 neutralizing antibodies (nAb) and at early onset (<72 h from symptoms onset), can block viral replication leading to a survival benefit [5,6].

Multiple mechanisms have been hypothesized to explain how CCP works against COVID-19. Specific antibody to SARS-CoV-2 is strongly implicated as an active agent in CCP based on dose-response clinical studies [7,8,9] and mechanistic studies that establish its antiviral activity [10]. Currently, nAb content is identified as the main driver of clinical benefit in CCP units. CCP includes a mix of over one thousand different serum proteins and chemical factors that may prove either therapeutic or detrimental for COVID-19 pathology. In this review, we analyzed factors where available preclinical or clinical evidence suggests a mediated effect on the clinical response to CCP.

## 2. Potential Beneficial Factors in CCP

In addition to anti-SARS-CoV-2 nAbs, several CCP components have been investigated as a possible explanation for the beneficial effect of CCP, including the role of immunomodulatory/anti-inflammatory, antithrombotic and direct antiviral properties of CCP.

### 2.1. Immunomodulatory and Anti-Inflammatory Properties of CCP

Besides the direct neutralizing effects of anti-spike IgG, IgG non-neutralizing antibodies present in CCP may also play a role in enhancing recovery in COVID-19 patients [11], mediated predominantly through their constant fragment (Fc), which has many known antimicrobial effects, including antibody-dependent cellular cytotoxicity (ADCC), antibody-dependent cellular phagocytosis (ADCP), and complement-dependent cytotoxicity (CDC) [12].

In addition to this immunomodulatory activity, a number of studies have consistently documented that administration of CCP is associated with lower levels of circulating cytokines such as tumor necrosis factor (TNF) and interleukin (IL)-6, thus reducing the detrimental hyperinflammatory response in COVID-19 patients [13,14]. Whether these effects result from viral neutralization with a consequent reduction in inflammation, a direct anti-inflammatory effect from a specific antibody, or attributable to non-immunoglobulin factors in CCP is uncertain. Several clinical studies have supported the anti-inflammatory properties of CCP [15]. A marked decrease of the proinflammatory markers C-reactive protein (CRP), ferritin, and lactate dehydrogenase (LDH) was observed 7 days after CCP transfusion in a proof of concept single-arm multicenter trial conducted in Italy on 46 severe COVID-19 patients [16]. Similarly, in a prospective cohort study conducted by Salazar and colleagues in 25 patients with severe or life-threatening COVID-19 [17], a marked reduction of CRP was observed at days 7 and 14 post-CCP transfusions. These results were replicated in other clinical trials [18,19,20]. Other studies compared the cytokine profile of CCP with that in plasma from healthy blood donors and found higher levels of IL-10, a potent anti-inflammatory cytokine, and IL-21, which is involved in plasma cell generation and antiviral immune responses [21].

The anti-inflammatory (and anticoagulant) activities of CCP can also be linked to the presence of major serine-protease inhibitors, particularly **alpha-1 antitrypsin (AAT)**, which is the most abundant serine protease inhibitor in plasma. AAT is a potent inhibitor of neutrophil elastase, thereby reducing pulmonary tissue damage and the formation of neutrophil extracellular traps. AAT has also been shown to exert anti-SARS-CoV-2 viral effects by inhibiting transmembrane serine protease 2 (TMPRSS2), a cell membrane-bound protease that promotes SARS-CoV-2 entry into host cells, and the disintegrin and metalloproteinase 17 (ADAM17). Therefore, it is conceivable that AAT in CCP exerts protective effects against COVID-19 infection, not only in patients suffering from congenital deficiency [22]. Plasma-derived AAT concentrates are currently under clinical evaluation in patients with COVID-19. However, a functional role for AAT in CCP has not yet been established.

### 2.2. Anti-Thrombotic Effect of CCP

Besides the involvement of the respiratory system, COVID-19 has been recognized as a systemic prothrombotic disorder [23]. The molecular mechanisms underlying the hypercoagulable state observed in patients with COVID-19 are not completely understood, although they presumably involve a close link between inflammatory and hemostatic systems. It is well known that SARS-CoV-2 infection produces endothelial dysfunction and a systemic inflammatory response leading to an imbalance between procoagulant and anticoagulant homeostatic pathways [11]. In particular, the elevated levels of proinflammatory cytokines (i.e., IL-1, IL-6, and TNF-alpha) induce an increased expression of tissue factor that, complexed with activated coagulation factor VII, initiates the extrinsic pathway of the coagulation cascade, leading to the formation of thrombin and conversion of fibrinogen into fibrin [23]. The concomitant hypofibrinolytic state, resulting from the viral-induced hyper-expression of plasminogen activator inhibitor-1, which directly inhibits tissue plasminogen activator (t-PA) and urokinase plasminogen activator (u-PA), creates a vicious circle that strengthens the thrombotic process [24].

Considering these reported effects of CCP, CCP appears to be a particularly appealing therapeutic tool to reduce pathology in COVID-19 patients, given that it contains the normal procoagulant and anticoagulant factors in a balanced physiologic ratio [5]. Additionally, several clinical studies have documented the anti-thrombotic effect of CCP, measured as a decrease in D-dimer levels, an important marker of thrombosis, and a worse prognostic indicator in severe COVID-19 patients [18,20,25]. In particular, CCP is a valuable source of some plasma proteins that play a key role in the hemostatic process, first of all, antithrombin and albumin [26,27].

- **Antithrombin III** is a universal constituent of donor plasma and works by improving the efficacy of heparin, which is one of the cornerstones of current COVID-19 management. Since AT-III levels are low in COVID-19 patients, it has been hypothesized that antithrombin III from CCP reduces the thrombotic risk in COVID-19 [26], but this has never been formally proven and no randomized controlled trial to date has reported a reduction in thrombotic events in the CCP arm;
- **Albumin** has been the object of intense research in the past few months. In an observational prospective cohort study, Violi and colleagues observed that albumin supplementation dampened hypercoagulability (measured as a reduction in D-dimer levels) in COVID-19 patients [28]. Similarly, a retrospective study by Kheir and colleagues found that higher albumin levels on admission were associated with a lower incidence of adverse outcomes, including venous thromboembolism (VTE), acute respiratory distress syndrome (ARDS) development, and intensive care unit (ICU) stay in COVID-19 patients [29].

### 2.3. Direct Antiviral Effects from CCP

- **Extracellular vesicles (EVs)** are also a universal component of donor plasma. EVs are lipid-bound vesicles secreted by cells into the extracellular space. The three main subtypes of EVs are micro-vesicles, exosomes, and apoptotic bodies. **ACE2-positive EVs** could act as decoy receptors since virions attaching to these EVs cannot complete a replicative cycle [30]. Recent experimental data show that ACE2-positive EVs can block SARS-CoV-2 spike-dependent infection [31]. EVs from plasma contain several other biomolecules such as miRNAs, proteins/cytokines, lipids, and glycan signatures that may alter the immune response to SARS-CoV-2 infection [32], but to date, a role for EVs in mediating the protective effect of CCP has not been demonstrated in vivo;
- **Coagulation factor Xa (FXa)** binds to and cleaves spike protein but produces a different cleavage pattern than that of furin and TMPRSS2, and, contrarily, what had been hypothesized initially [33], blocks S protein binding to ACE2. The effect was pronounced for the ancestral wild-type variant but was diminished in the B.1.1.7 variant. Exogenous FXa protected mice from lethal infection in a humanized hACE2 mouse model of COVID-19 using the wild-type variant but not the B.1.1.7 variant. The antiviral effect of FXa was attenuated by the direct FXa inhibitor rivaroxaban but not the indirect inhibitor fondaparinux, both in vivo and in vitro [34];
- **Cross-reactive neutralizing antibodies**: The basis for heterologous immune responses to SARS-CoV-2 is likely due to cross-reactivity between the surface antigens. Antigenic cross-reactivity can derive from previous exposure to a variety of …
  - pathogens
    ○ **Seasonal coronaviruses** [35]: Patients with severe COVID-19 had significantly lower levels of OC43 and HKU1 [36] or significantly higher NL63 and 229E [37] nucleoprotein-specific antibodies compared with other COVID-19 patients. The prognostic role of low OC43 antibodies was confirmed in another study: OC43 negative inpatients had an increased risk of severe disease (adjusted odds ratio 2.8), higher than the risk conferred by increased age or body mass index, and lower than the risk by male sex [35]. These findings may also imply convalescent plasma collections (e.g., CCP units with greater NL63 antibody responses and lower HKU1 antibodies) had higher neutralizing antibodies to the SARS-CoV-2 receptor-binding domain (RBD) [38]. Another study found better outcomes in recipients of CCP units with higher anti-NL63 or anti-OC43 antibodies [39];
    ○ **Influenza virus A(H_3_N_2_)**: Antibody binding to an epitope region from SARS-CoV-2 nucleocapsid, termed Ep9, is associated with greater COVID-19 disease severity [40]. Bioinformatics analysis identified the neuraminidase protein (not present in the influenza vaccine) of influenza virus A(H3N2) as responsible, a strain that circulated widely in 2014 [41];
    ○ Acute **malaria** infection: *Plasmodium* infection induces cross-reactive antibodies to carbohydrate epitopes on the SARS-CoV-2 spike protein [42];
    ○ **Natural ABO isoagglutinins:** The ABO blood group affects COVID-19 incidence and severity, as well as the type and duration of the cellular immune response [43]. Analogous to the events of SARS-CoV-1, it was hypothesized that natural isoagglutinins act as neutralizing antibodies owing to ABO antigens being carried over on virion envelope [44], although the evidence to date is weak [45].
  - vaccination:
    ○ **MMR** (measles-mumps-rubella) or **Tdap** (tetanus-diphtheria-acellular pertussis) vaccination [46]. Of interest, the SARS-CoV-2 spike protein displays biologically significant amino acid sequence similarities with paramyxovirus surface proteins [47]. A significant inverse correlation between mumps titers from MMR II and COVID-19 severity has also been reported [48];
    ○ **Influenza vaccination:** Among 472,000 cases in Brazil, regression analysis showed an almost two-fold odds ratio for invasive ventilation, Intensive care unit (ICU) admission, and death in unvaccinated cases [49].

## 3. Potential Detrimental Factors in CCP

Numerous factors in plasma can either be of no benefit or drive immunopathology following SARS-CoV-2 infection, be present prior to infection, or increasing in concentration during COVID-19. When considering the latter scenario, plasmapheresis has been proposed as a therapeutic approach either per se or followed by CCP treatment [50]. In addition to the beneficial factors listed in the previous section, these detrimental factors are likely found in donor CCP.

### 3.1. Direct Proviral Effect

- **Spike-activating serine endoproteases** can act as surrogates for TMPRSS2 at cleaving SARS-CoV-2 spike protein at the so-called furin cleavage site (FCS), creating S1 and S2 subunits. **Thrombin** is an endoprotease that increases SARS-COV-2 cell entry in vitro via this mechanism [33]. Since this enhances viral entry, more proteases can lead to more infection, but this has not been formally proven in vivo. A model of positive feedback was proposed whereby infection-induced hypercoagulation exacerbates SARS-CoV-2 infectivity. Anticoagulation is hence critical in managing COVID-19, and early intervention may provide collateral benefit by suppressing SARS-CoV-2 viral entry [33];
- **Virus-carrying EVs**: Despite SARS-COV-2 RNA viremia being extremely low and transient, SARS-CoV-2 RNA has been detected inside EVs [51]. Compared to the hyperinflammatory phase, EVs from the resolution phase induce opposing effects on eukaryotic translation and Notch signaling [52]. However, it is unclear whether these occur in recovered CCP donors and their infectious potential has not been established [53]. This concern represents an indication for applying pathogen reduction technologies to therapeutic CCP.

### 3.2. Pro-Coagulant Factors

Regular donor plasma includes physiological levels of both pro-coagulant and anti-coagulant factors. Since COVID-19 is a prothrombotic disorder leading to the consumption of pro-coagulant factors, replacing these factors with new ones provided by CCP may fuel thrombosis, theoretically promoting pulmonary thromboembolism [54]. However, it is noteworthy that a unit of CCP is a small fraction of the circulating plasma volume. The amount of pro-coagulant and anti-coagulant factors delivered during one 200 mL transfusion is small relative to the physiologic needs of an ongoing pathogenic process that consumes proteins involved in the coagulation cascade. Nevertheless, large case series are reassuring regarding the low risk for thrombotic complications after CCP transfusion [55].

- **Tissue factor expressing EVs** [56] are found in blood circulation, and their level parallels the intense thrombo-inflammatory state and thrombosis observed in severe COVID-19. However, we are not aware of studies examining the content and type of EVs in CCP. Clinical data using CCP did not identify a higher risk of thrombotic events suggesting that pro-coagulant tissue factors expressing EVs disappear quickly from the blood circulation upon resolution of the symptoms;
- **Anti-ADAMTS13 autoantibodies**: Doevelaar et al. reported antibodies to ADAMTS13 in 31 (34.4%) patients with COVID-19. Generation of ADAMTS13 antibodies was associated with a significantly lower ADAMTS13 activity, increased disease severity (a severe or critical disease in 90% vs. 62.3%), and a trend to higher mortality (35.5% vs. 18.6%). The median time to antibody development was 11 days after the first positive SARS-CoV-2-PCR specimen [57];
- **Antiphospholipid (aPL) antibodies**: A meta-analysis of over 1159 patients reported that aPL antibodies were detected in nearly half of patients with COVID-19, with a higher prevalence of aPL found in those with severe disease. However, there was no association between aPL positivity and disease outcomes, including thrombosis, invasive ventilation, and mortality [58]. **Anti-cardiolipin IgA and IgM, and anti-β2 glycoprotein-1 IgA** were found in 5–12% of hospitalized patients [59,60] and were elevated in severe COVID-19 [61]. Nevertheless, Borghi et al. reported that aPLs show a low prevalence in COVID-19 patients and are not associated with major thrombotic events. The aPLs in COVID-19 patients are mainly directed against β_2_-GPI but display an epitope specificity different from antibodies in aPL syndrome [62]. **Lupus anticoagulant (LA)**, a misnomer for prothrombotic antibody, was found in 46.6% of hospitalized COVID-19 patients, but no association was found with mortality or the need for mechanical ventilation in survivors [63]. Most importantly, LA is transient, but other aPLs are persistent [64] and potentially found in CCP donors. **Anti-prothrombin antibody** levels are associated with disease severity and anti-SARS-CoV-2 antibody levels [65];
- **Autoantibodies against annexin A2**, which are known to induce systemic thrombosis, cell death, and non-cardiogenic pulmonary edema, were elevated among 86 hospitalized COVID-19 patients and predicted mortality (OR = 9.3) [66];
- **α(2)-antiplasmin (α2AP), various fibrinogen chains, and Serum Amyloid A (SAA)** are substantially increased and trapped in the solubilized fibrinolytic-resistant pellet deposits found in plasma from patients experiencing long COVID-19/post-acute sequelae of COVID-19 (PASC) syndrome. Albeit, such patients are unlikely to donate CCP and mild presentations could pass screening visits [67];
- **Soluble urokinase-type plasminogen activator receptor (sUPAR)** is highly expressed by an abnormally expanded circulating myeloid cell population in severe COVID-19 patients with ARDS [68]. Plasma sUPAR level was found to be linked to a characteristic proteomic signature of plasma, linked to coagulation disorders, and complement activation.

### 3.3. Proinflammatory or Immunosuppressive Factors

- **Afucosylated IgG** defines an exacerbated phenotype in COVID-19: afucosylated immune complexes in the lungs trigger an inflammatory infiltrate and cytokine production dependent on the expression of the receptor for afucosylated IgGs, FcγRIIIa (CD16a) in monocytes [43]. Accordingly, elevated frequencies of CD16a+ monocytes were another antecedent in patients with more severe outcomes [43]. Immune complexes contained recombinant SARS-CoV-2 spike protein and aberrantly glycosylated anti-spike IgG with enhanced platelet-mediated thrombosis on von Willebrand Factor in vitro [69];
- **Autoantibodies**: SARS-CoV-2 infection can trigger autoimmune diseases such as myocarditis, and many single cases have been reported in the literature. In this review, we focus on large case series that help assess the prevalence of autoantibodies.
  ○ **Autoantibodies against interferons (IFNs)** are commonly found in 40% of systemic lupus erythematosus patients. Bastard et al. identified nAb against type I IFN-α2 and IFN-ω in about 10% of patients with severe COVID-19 pneumonia, but not in patients with an asymptomatic or mild disease [70]. This cohort has the highest likelihood of having antiviral nAbs [71] and hence more likely to be selected as CCP donors. Accordingly, Vazquez et al. found nAbs to IFNs in 3% (4/116) of CCP donors [72]. By neutralizing one of the key mediators of the effector arm of the immune response, these antibodies may function as immune suppressants, which could help or hurt the recipient patient depending on the stage of the disease;
  ○ **Autoantibodies against melanoma differentiation-associated gene 5 (MDA5)** characterizes a subtype of dermatomyositis (DM) and were found in 48.2% (132/274) of COVID-19 patients. The anti-MDA5 Ab positive patients tended to represent severe COVID-19 (88.6% vs. 66.9%). The titer of Ab to MDA5 was significantly elevated in non-surviving patients, and the positive rate was also higher than in survivors (23.5% vs. 12.0). With regards to those patients with severe COVID-19, high titers of Ab to MDA5 (≥10.0 U/mL) were more prevalent in those who did not survive (31.2% vs. 14.0%) [73]. Of interest, both MDA5-associated DM and COVID-19 can involve the lungs, skin, and skeletal muscles. The initial radiological features of lung pathology in DM patients with Abs to MDA5 are mainly subpleural ground-glass opacities or mixed with consolidation and signs of acute respiratory distress syndrome (ARDS) resembling severe and critical COVID-19. MDA5 is a crucial cytoplasmic sensor for viral RNA, and its expression is induced by RNA viruses (including SARS-CoV-2 [74]). Viral infection activates the expression of antiviral type I and III interferons (IFNs) and other inflammatory cytokines;
  ○ **Antinuclear autoantibodies (ANA)** were found in 11–57% [59,60,75] of hospitalized COVID-19 patients. Woodruf et al. identified ANA in 44% of 31 critically-ill patients with COVID-19 with no known history of autoimmunity [76]. Specifically, Gomes et al. showed that antibodies to DNA determined hospital admission and correlated strongly with the later development of severe disease, showing a positive predictive value of 89.5% and accounting for 22% of total severe cases [77]. Anti-extractable nuclear antigen (ENA) antibodies were reported in 2.5% of hospitalized COVID-19 patients [60];
  ○ **Antineutrophil cytoplasmic autoantibodies (ANCA)** were found in 6.6% of hospitalized COVID-19 patients [75] but were absent in a different series of 33 patients [59];
  ○ **IgM autoantibodies against ACE2** (the cellular receptor for SARS-CoV-2 spike protein) were detected in 27% of 66 severe COVID-19 patients vs. 3.8% of 52 non-hospitalized patients [78]. If and how they contribute to angiocentric pathology remains unknown. The antibodies do not undergo class-switching to IgG, suggesting a T cell-independent antibody response. Purified IgM from anti-ACE2 patients activates complement;
  ○ **Autoantibodies against angiotensin II type 1 receptor (AT1R)**: No statistically significant differences were found between COVID-19 cases and controls. However, there were trends toward a higher proportion with AT1R autoantibody positivity among severe cases versus controls (32% vs. 11%) and higher levels in those with mild COVID-19 compared with controls (median 9.5 U/mL vs. 5.9 U/mL [79]);
  ○ **Autoantibodies against anti-malondialdehyde (MDA) and anti-adipocyte-derived protein antigens (AD)** are more frequent in lean than in obese COVID-19 patients compared to uninfected controls. However, serum levels of these autoantibodies are always higher in obese versus lean COVID-19 patients and associated with CRP levels [80];
  ○ **Anti-neuronal or anti-glial autoantibodies** (e.g., **against Yo or NMDA receptor)**, which theoretically crossed a leaky brain-blood barrier, were universally detected in plasma and cerebrospinal fluid of 11 severely ill COVID-19 patients presenting unexplained neurological symptoms [81].

These findings suggest that studies should be conducted to determine whether the plasma of individuals harbors multiple autoantibodies following SARS-CoV-2 infection and whether these are related to the increased prevalence of autoimmune diseases or immune-complex mediated pathology. Infections have long been postulated to play a role in causing or promoting autoimmune diseases, and COVID-19 may provide some of the clearest evidence for their role.

## 4. Conclusions

**Figure 1:**
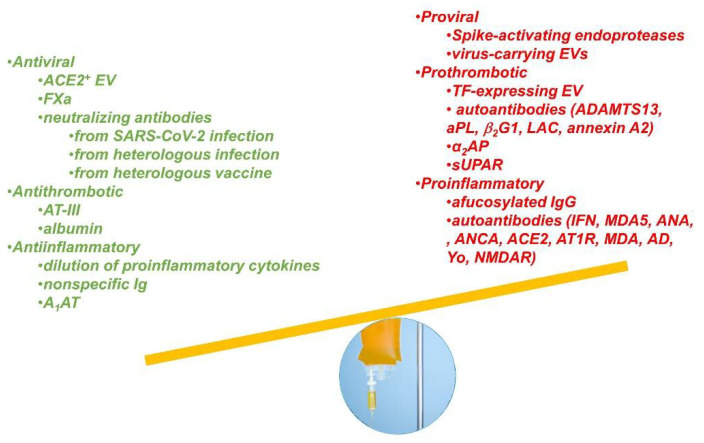
Visual representation of the different beneficial and detrimental co-factors potentially contained in a convalescent plasma unit. EV—Extracellular vesicles; A1AT—Alpha1-antitrypsin; TF—Tissue factor.

A logical conclusion from available data is that CCP, even collected months after resolution of infection, may contain many biological factors which, once transfused, may have the potential to influence the outcome of COVID-19 (Figure 1). Furthermore, the available evidence from controlled studies is that CCP therapy is found to either have no effect or improve outcomes from COVID-19. Thus, any negative effects of CCP therapy in patient outcomes are likely to be small or rare given the absence of any significant toxicity reports [55].

Including standard fresh frozen plasma for control arms should be considered in future clinical studies involving CCP. Such inclusion and analysis of contents can be achieved in small- to medium-scale RCTs and should not be considered wasting precious resources; on the contrary, they are the only evidence-based method to formally identify which active ingredients in CCP are more important for delivering clinical benefit. However, the use of non-convalescent plasma in the control arm will not discriminate those factors found only in CCP, such as EVs elicited directly as part of the immune response and pathogenic process to SARS-CoV-2. Establishing a causal link between the presence of many non-Ab components found in the CCP described in this review that may affect the therapeutic outcome remains a formidable problem. To accomplish this, their effects must be separated from those of specific antibodies present in larger quantities due to the convalescent immune response and the selection of high titer units.

Hence, three-arms RCT including best supportive care (BSC), BSC plus non-convalescent fresh frozen plasma, and BSC plus CCP should help discern if factors other than nAb in CCP impact clinical outcomes. At this stage of the pandemic and with massive deployment of vaccine campaigns, running such trials may, however, prove difficult but can be a lesson for future pandemics.

More pragmatically, the best approach to determining whether some of these biological factors matter may be to retrospectively study situations where patients performed disproportionately better or worse than expected based on the nAb titer and then analyze the remaining aliquots of the infused plasma for the various components described in this review. However, given a large number of non-antibody components in CCP, the variable nature of COVID-19, and the possibility that these factors act in combination, establishing causality for any of these components may require very large studies.

In a situation where clarity on the contribution of non-Ab components to CCP efficacy is unlikely to be forthcoming in the near future, physicians and investigators must be aware of potential confounders in therapeutic studies and maintain a high index of alertness for unusual responses to CCP therapy. These should be investigated in detail since they might provide important hints as to whether the other plasma co-factors are important for COVID-19 outcomes.

## Data Availability

All data present in the text derive from articles published elsewhere and were accessible via references.
